# Fabrication of chitosan-polyvinyl alcohol and silk electrospun fiber seeded with differentiated keratinocyte for skin tissue regeneration in animal wound model

**DOI:** 10.1186/s13036-020-00249-y

**Published:** 2020-11-18

**Authors:** Afshin Fathi, Mehdi Khanmohammadi, Arash Goodarzi, Lale Foroutani, Zahra Taherian Mobarakeh, Jamileh Saremi, Zohreh Arabpour, Jafar Ai

**Affiliations:** 1 Department of Plastic and Reconstructive Surgery, School of Medicine, Tehran University of Medical Sciences, Tehran, Iran; 2 Skull Base Research Center, The Five Senses Institute, Hazrat Rasoul Akram Hospital, Iran University of Medical Sciences (IUMS), Tehran, Iran; 3 Department of Tissue Engineering and Applied Cell Sciences, School of Advanced Technologies in Medicine, Fasa University of Medical Sciences, Fasa, Iran; 4 Tehran University of Medical Sciences, Tehran, Iran; 5 Department of Tissue Engineering and Applied Cell Sciences, School of Advance Technologies in Medicine, Tehran University of Medical Sciences, Tehran, 1417743361 Iran; 6 Department of Biomedical and Electronics Engineering, School of Engineering, University of Bradford, Bradford, UK; 7 Brain and Spinal Cord Injury Research Center, Neuroscience Institute, Tehran University of Medical Sciences, Tehran, Iran

**Keywords:** Hybrid fiber, Chitosan, Poly vinyl alcohol, Silk, Mesenchymal stem cells, Keratinocytes, Electrospinning, Skin tissue regeneration

## Abstract

Hybrid fibrous mat containing cell interactive molecules offers the ability to deliver the cells and drugs in wound bed, which will help to achieve a high therapeutic treatment. In this study, a co-electrospun hybrid of polyvinyl alcohol (PVA), chitosan (Ch) and silk fibrous mat was developed and their wound healing potential by localizing bone marrow mesenchymal stem cells (MSCs)-derived keratinocytes on it was evaluated in vitro and in vivo. It was expected that fabricated hybrid construct could promote wound healing due to its structure, physical, biological specifications. The fabricated fibrous mats were characterized for their structural, mechanical and biochemical properties. The shape uniformity and pore size of fibers showed smooth and homogenous structures of them. Fourier transform infrared spectroscopy (FTIR) verified all typical absorption characteristics of Ch-PVA + Silk polymers as well as Ch-PVA or pure PVA substrates. The contact angle and wettability measurement of fibers showed that mats found moderate hydrophilicity by addition of Ch and silk substrates compared with PVA alone. The mechanical features of Ch-PVA + Silk fibrous mat increase significantly through co-electrospun process as well as hybridization of these synthetic and natural polymers. Higher degrees of cellular attachment and proliferation obtained on Ch-PVA + Silk fibers compared with PVA and Ch-PVA fibers. In terms of the capability of Ch-PVA + Silk fibers and MSC-derived keratinocytes, histological analysis and skin regeneration results showed this novel fibrous construct could be suggested as a skin substitute in the repair of injured skin and regenerative medicine applications.

## Introduction

Scaffolds are bioactive substrates that play vital role in tissue repair and regeneration since they could mimic components and structural perspectives of extracellular matrix (ECM) [1–4]. Scaffolds should not only possess suitable mechanical properties, but also need to imitate the structural aspects of natural ECM to create an ideal support for cell seeding, adhesion, proliferation as well as differentiation with least inflammatory and toxic reactions [1–3, 5–7]. Natural ECM consists of diverse interwoven protein fibers with nanometer diameters and nanoscale structure [1, 2, 6, 8]. These nanoscale structures can support cell functions and direct cell fate [1, 2, 6]. In this regard, fabricating scaffolds with the same architecture of native tissues is one of the main challenges in this field [1, 2, 6, 8]. Production of nanoscale scaffolds as ECM substitutes have been well developed in various methods including phase separation, self-assembly, synthetic molding, microfluidic and electrospinning [1, 2, 6, 9]. Among these, the electrospinning process has recently gained considerable attention because of its high degrees of processability, diverse applicability of biopolymers over other methods and capacity in mimicking extracellular matrix (ECM) structure with adjustable porosity and pore size distribution of fibers [1, 2, 6, 10]. Furthermore, the large surface area of electrospun fibers and porous structure can extensively enhance cell viability and functionality [1, 2, 6, 11, 12]. Until now, the wide range of polymers are capable of being electrospun which shows flexibility in designing fibrous scaffolds from pure or blended of natural and synthetic polymers [1, 2, 6, 8, 11–13]. Natural polymers include collagen, fibrin, silk, carboxymethyl cellulose (CMC), hyaluronic acid (HA) and chitosan (Ch) which are actively interacted with cells through cell surface receptor ligands and in following cell-signaling pathways, but they are expensive, not easy accessible and have poor mechanical properties [1, 13–16]. By contrast, synthetic polymers provide great applicability by chemical or physical modifications and their excellent processability [1, 6, 16, 17]. However, these polymers lack bioactivity and special care needs to be taken to ensure that newly synthesized polymers are biocompatible [1, 6, 16, 17]. Poly vinyl alcohol (PVA) is food and drug administration (FDA) approved polymer for clinical use due to its degradability, cytocompatibility and processability as well as excellent strength and elongation properties [1, 5, 6, 8, 18]. Nevertheless, single-component biopolymer is generally insufficient for good physical and biochemical fiber specifications [2, 14, 16, 19–21]. To overcome these limitations, recent effort has been given to takes advantage of the physical properties of the synthetic polymers and the bioactivity of the natural polymers while minimizing disadvantages of both combine for the preparation of electrospun fibers [2, 14, 16, 19–21].

Silk protein generated from silk cocoons has been extensively used for the medical applications including wound healing, tissue regeneration and drug delivery [5, 10, 22]. Silk derivatives contain high quantity of (Gly-Ala-Gly-Ser) n sequences in the crystalline region, (Ala) n sequences as well as Arg-Gly-Asp tripeptide, which may function as a biological recognition signal and encourage cell adhesion [21–23]. Besides, Ch derivative as a biomimetic polymer owing to its hemostatic, stimulation of healing, antimicrobial, nontoxic, biocompatible and biodegradable properties has received significant status on wound healing and skin tissue regeneration [5, 6, 19]. Cell adhesion to Ch hydrogels and their degradation can be controlled by N-acetylation. The N-acetylglucosamine moiety in Ch is a structural feature also found in glycosaminoglycan (GAG), a component of native ECM. Since the properties of GAG include specific interactions with bioactive components and cells, this suggests that the analogous structure of Ch may mimic these bioactivities. It is expected that with combination of synthetic PVA and natural derived polymers including Ch and silk could provide proper microenvironment for cell proliferation and differentiation in skin regeneration.

**Fig. 1:**
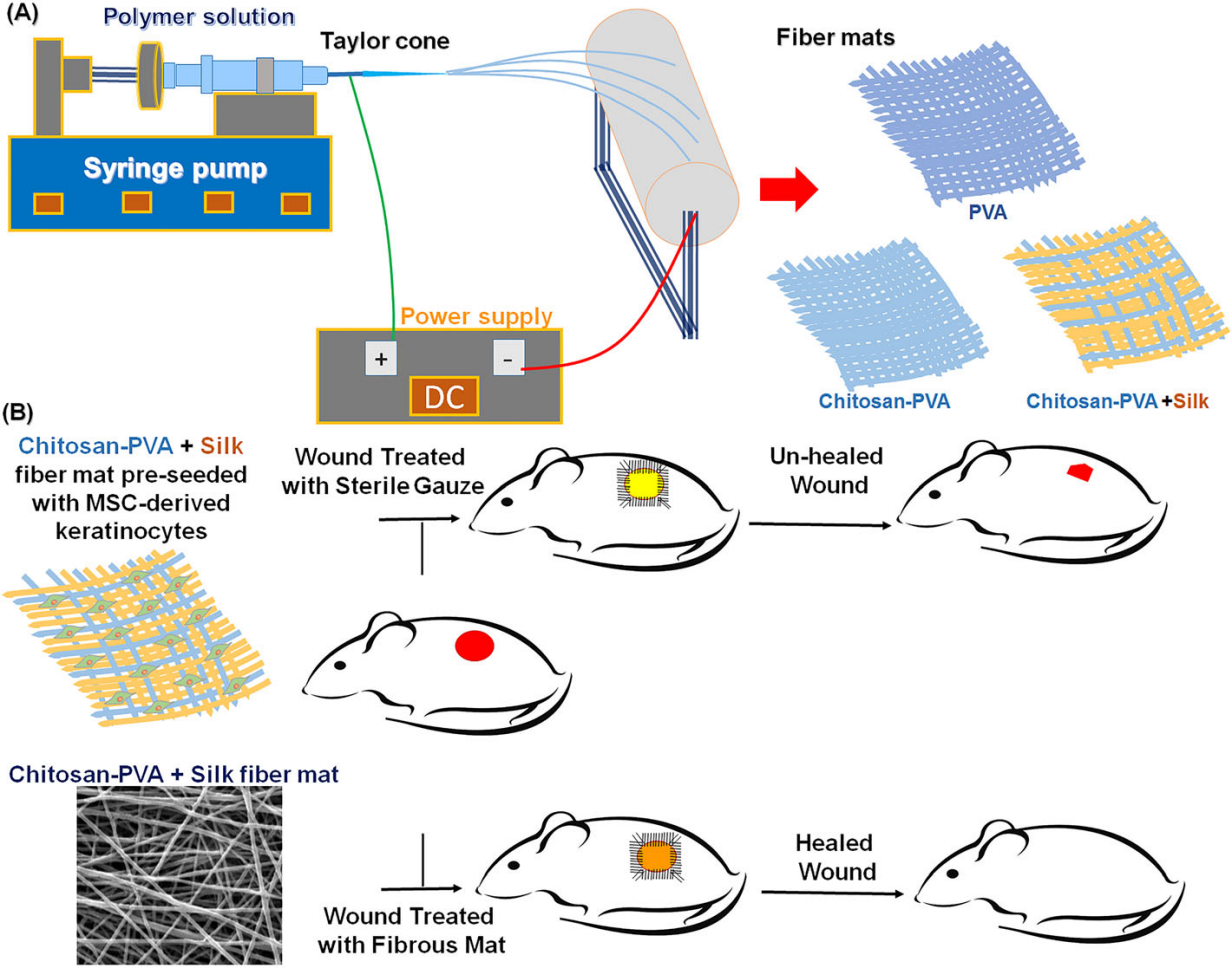
Schematic representation showed (**a**) hybrid chitosan (Ch), polyvinyl alcohol (PVA) and silk fiber mat fabrication through electrospinning method and (**b**) its utilization in full-thickness wound 6 excision rat model to evaluate wound healing potential of transplanted Ch-PVA + Silk fibrous mat pre-seeded MSC-derived keratinocytes

In this study we have fabricated hybrid Ch-PVA + Silk fibrous mat in uniform size and desirable porosity, degradability and mechanical properties through co-electrospinning process for wound healing application in a full-thickness excisional animal model. The physical and chemical characteristics of Ch-PVA + Silk fibrous mat including pore size, porosity, tensile strength, degradability and hydrophilic properties were evaluated in vitro. The cellular attachment, morphology and proliferation on fabricated fibers were investigated during extended time of incubation. The differentiation potential of mesenchymal stem cell to keratinocyte performed using defined conditional media. The wound healing test involved using Ch-PVA + Silk fibrous mat seeded with MSC-derived keratinocytes was performed in rat animal models (an overview of this study is shown in Fig. 1).

## Materials and methods

### Materials

Ch (low in molecular weight (Mw), degree of deacetylation 75–85%) was obtained from Shanghai Hanshare Industry Co., Ltd. Silk was obtained by following previous reported protocols [22]. Briefly, cocoons of mulberry *Bombyx mori* silkworm were heated in 0.5% (w/v) sodium carbonate within 1 h followed by washing in distilled water through centrifugation at 4000 rpm for 10 min which help to remove debris. Degummed fibers were dried at room temperature and these fibers were dissolved in 9 M of lithium bromide solution at 60 °C. The aqueous polymeric solution was dialyzing against deionized water and finally dried at 0.074 mb and − 53 °C for 2 days. The PVA (Mw: 85,000-124,000, 87–89% hydrolyzed), and all solvents, reactants and dialysis membrane (Mw: 12000 Da) were purchased from Sigma-Aldrich.

### Preparation of solvent and electrolyte solution PVA

PVA solution of 10% (w/v) was prepared by dissolving in distilled water at 85 °C. The Ch was also dissolved in 0.01 M acetic acid pH 4 at a concentration of 2% (w/v) under stirring for 1 h. The silk solution of 10% (w/v) was obtained by dissolving its powder in hexafluoroisopropanol 50% (w/v). Then, PVA and Ch solutions were combined in a volume ratio of 9: 1 under magnetic stirring at ambient temperature to form a stable suspension. Then, the pure PVA or Ch-PVA mixture and silk solutions were electrospun (electrospray device; Gene Fanavaran Nanomeghyas, Iran) by 5 ml syringe connected to stainless steel 21-gauge blunt ended needle in reverse direction at room temperature. The electrospinning factors were selected counter current co-electrospun process as follows: discharge rate for both solutions: 0.4 mL/h, voltage for PVA or Ch-PVA solution: 18 KV and for silk solution: 14KV, the distance between the tip of the needles and rotating wheel covered by aluminum foil for PVA or Ch-PVA solution: 20 cm and for silk solution: 15 cm. To concentrate the fibers on the collector surface, other parts of the machine were insulated except for the place where the fabricating nanofibers were to be assembled by aluminum foil. The co-electrospinning process was stopped after 6 h running and electrospun fibrous sheets were crosslinked by contact with 50% glutaraldehyde in desiccator overnight. The fibrous mats were vacuumed for 2 h to remove any remained solvent before usage. The morphology of the initial electrospun fibers was investigated by conventional light microscopy for regular and nodal arrangement.

### Characterization of electrospun scaffolds

The morphology and average diameter of fibrous mats were evaluated by a scanning electron microscopy (SEM, Philips XL30: Eindhoven, The Netherlands). The fabricated scaffolds were surface coated by gold layer using a sputter coater machine (Hummer 62, Ladd Research). The average diameter of the fabricated scaffolds was evaluated by image analysis software (Image J, NIH). Functional groups of fabricated fibrous scaffolds were evaluated using fourier transform infrared spectroscopy (FTIR). The FTIR spectroscopy was performed on a Bruker instrument (Aquinox 55, Germany) in the range of 400–4000 cm^− 1^ and with a resolution of 1 cm^− 1^. Distribution of prepared fibrous mat pores, porosity percentage and mean value of pore diameter was measured using mercury porosimetery. The change in swelling ratio as well as hydrophobicity of fabricated PVA based fibrous mats were evaluated by the swelling test and contact angle analysis. The fibrous scaffolds were cut and weighed at 1 × 1 cm to evaluate the water uptake. Each sample was removed after 24 h immersion in simulated liquid (PBS, Gibco) and gently placed between two filter papers to remove excess water. The samples were then weighed and the degree of swelling or water absorption of each sample was calculated by the following formula:

Degree of swelling (%) = (W_st_ -W_dt_) / w_dt_ × 100.

W_st_ is the wet weight after immersion in PBS and W_dt_ is the sample weight in the dry state before immersion. The experiment was repeated three times and the results were averaged. The degradability of fibrous mats was evaluated during extended time of incubation for swelled mats. The scaffolds were placed in 6 mL PBS and incubated at 37 °C for 14 days. At specified intervals the samples were taken out from the buffer. The samples were washed with deionized water at room temperature to remove the dissolved inorganic salts. Then, those were then weighed (W_t_). The weight loss (W_L_) of the scaffold was calculated using the following formula for water absorption where W_0_ is swelled fiber weight.

$$ {\mathrm{W}}_{\mathrm{L}}\%=\left({\mathrm{W}}_{\mathrm{t}}-{\mathrm{W}}_0\right)/{\mathrm{W}}_0\times 100 $$

Mechanical properties of the fibrous mats were measured by the tensile strength evaluation. To this, the dry rectangular sample mats were cut in 4 × 1 cm^2^ in dimensions and fixed between the grips of the testing device (Santam, Karaj, Iran). The mats were stretched in the axial direction with the rate of 5 mm/min and the ultimate tensile strength was obtained for samples.

### Isolation and culture of bone marrow mesenchymal stem cells

NMRI mice aged 5 to 6 weeks were used for isolation and culture of bone marrow mesenchymal stem cells (bMSCs). The Mice were first scarified by cervical spine removal. The both humerus and tibia were soaked in hank’s balanced salt solution (HBSS; Sigma, H6648) supplemented with 15% (v/v) fetal bovine serum (FBS, Atlanta Biologicals) and penicillin/streptomycin (100 U mL^− 1^, 0.1 mg mL^− 1^). Then bones were cut and bone marrow was removed by dulbecco’s modified eagle’s medium (DMEM) using a flushing insulin syringe. The isolated cells were placed into a 15 mL conical tube and after centrifugation, the bMSCs were cultured in a 25 cm tissue culture flask containing DMEM supplemented with 20% FBS and antibiotics at 5% CO_2_ and 37 °C. The cell culture media was replaced every 3 days.

### Characterization of keratinocyte

The harvested cells 1 × 10^5^ cells at passage 3–4 were incubated with specific monoclonal antibodies tagged with FITC directed against CD31, CD34, CD73, or CD105 for 30 min at room temperature. Stained cells were rinsed with phosphate buffered saline (PBS; pH: 7.4) and were fixed in 1% formaldehyde solution and studied using BD FACCS Calibur (BD bioscience San Jose, CA, USA). Furthermore, the differentiation potential of adherent fibroblastoid cells into keratinocyte lineage was performed as described previously [24, 25]. For this, cells at density 1 × 10^5^ were seeded in 6-well tissue plate and incubated in conditional DMEM media containing 2 mM calcium chloride, 5 μg/mL insulin and 10 ng/mL recombinant human epidermal growth factor (EGF) and keratinocyte growth factor (KGF) when changing culture media every 3 days until 18 days. The cultured cells were evaluated via immonohistochemical staining after fixing with 4% formaldehyde. The cells were treated with specific keratinocyte antibodies for 15 min and after that stained samples were imaged under LABOMED TCM 400 microscope.

### Cell attachment and proliferation on Ch-PVA and silk fibrous mat

The Ch-PVA and silk fibrous scaffold 1 × 1 cm in size was immersed in 70% ethanol for 1 h. To assure sterilization of fibrous scaffold, each side of sample was exposed ultraviolet light at 245 nm for 30 min. The fibrous scaffold was then placed in 24-well tissue plate and were washed with PBS buffer. The MSC-derived keratinocytes were seeded at 1 × 10^4^ cells on fibrous scaffold surface and incubated for 4 h to allow the attach cells on scaffold structure. The cellular attachment was evaluated by SEM analysis. To this, fibers with seeded MSC-derived keratinocytes were washed with PBS and cells were fixed on the these using 2.5% (w/v) glutaraldehyde for 4 h. Then, fibrous scaffolds were dehydrated through a graded ethanol series (50 to 95% (w/v)), and then sputter-coated with gold. The cellular morphology was examined using a scanning electron microscope (SEM; Philips XL30, Netherland). Moreover, for qualitative evaluation of cell viability, cell seeded on fibrous scaffolds were treated with DAPI (4′,6-diamidino-2-phenylindole) solution and incubated in dark condition for 15 min. Then, samples were washed twice with PBS to remove non-reacted DAPI solution and examined by fluorescent microscopy.

### Cellular proliferation on Ch-PVA and silk fibrous mat

Cellular growth was evaluated by 3-[4,5-dimethyl-2-thia-zolyl]-2, 5-diphenyl-2H-tetrazolium bromide (MTT) assay as well as DAPI staining for seeded MSC-derived keratinocytes on fibrous mats during extended time of incubation. For this, 0.5 mL DMEM media and 50 μL MTT reagent were added to the samples and incubated for 4 h at 37 °C. The mitochondrial enzyme succinate-dehydrogenase within viable cells cleavages tetrazolium salt MTT into a blue-colored product (formazan). The formazan derivatives in medium was dissolved in 0.5 mL DMSO solution. The absorbance of each sample was measured using a spectrophotometer at 570 nm. The quantity of formazan produced is correlated with the number of living cells in the sample.

### Wound healing model

Twelve male Wister rats (average weight 200–250 g) were used for wound healing analysis. Animal experiments were carried out in compliance with the National Institutes of Health (NIH Publication No. 8023, revised 1978) guidelines for the care and use of laboratory animals and approved by the local Ethics Committee of “Regulations for using animals in scientific procedures in Tehran University of Medical Sciences. Rats were anesthetized with intraperitoneal injection of ketamine 5%/Xylazine 2% (Alfasan Co., Woerden, Netherlands) and dorsal skin was shaved and cleaned with betadine solution. Full-thickness circular wound (including panniculus carnosus) was inflicted with 10 mm dermal biopsy punches on site of the shaved back. The wounds were washed with physiologic serum and the rats were randomly divided into three groups (*n = 4* in each group): paraffin sterile gauze, Ch-PVA + Silk, and Ch-PVA + Silk + MSC-derived keratinocyte groups. The density of MSC-derived keratinocytes was 2 × 10^5^ cells which seeded on fibrous mat for 4 h prior suturing fibrous on wound site. The fiber scaffolds were sutured on wound sites using synthetic suture material and elastic adhesive bandage to fix the dressings. On day 14 post-surgery, all the wounds in each group were washed with physiologic serum and the animals were sacrificed for further analysis.

### Histopathology study

Animals were euthanized 14 days’ post-treatment and the skin tissues were harvested and immediately fixed in the 10% neutral buffered formalin (PH. 7.26) for 48 h. Then the fixed tissue samples were processed by embedding in paraffin sheet, and sectioning in 5 μm thickness. Finally, the sections were stained with haematoxylin and eosin (H&E) and Masson’s trichrome (MT). The histological slides were evaluated by the independent reviewer, using light microscopy (Olympus BX51; Olympus, Tokyo, Japan) and analyzed using image j software. Epithelialization, inflammatory cell infiltration, fibroplasia, and granulation tissue formation have assessed in different groups, comparatively. Magnification × 400 was employed for counting the cells and the calculation was repeated for six fields.

### Histomorphometry analysis

Epithelialization was assessed semiquantitatively on 5 point scale: 0 (without new epithelialization), 1 (25%), 2 (50%), 3 (75%), and 4 (100%). Moreover, the number of inflammatory cells and newly formed blood vessels were recorded in each group. For these parameters, results were validated by a comparative analysis of one independent observer blinded to the treatment groups.

### Statistical analysis

All experiments were carried out in triplicate for each condition. The data are shown as means ± standard deviation (StDev). Statistical analysis was performed by Minitab 18 software (Minitab, Inc., State College, USA). Significant differences were shown as *P-value < 0.05, **P-value< 0.01, whereas ns show *P-*value > 0.05.

## Results

### Morphology and structure of Ch-PVA and silk fiber

**Fig. 2:**
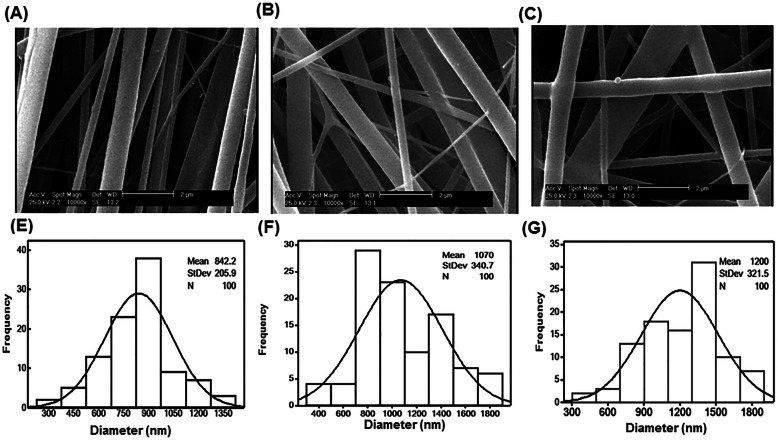
SEM photograph (**a**, **b**, **c**) and normal size distribution (**e**, **f**, **g**) of nanofibers in different composition PVA (**a**, **e**), Ch-PVA (**b**, **f**), and Ch-PVA + Silk (**c**, **g**)

Polymer solutions were continuously extruded from the tip of the needles in co-electrospinning process while drawing the polymer solutions towards the collector continues under the influence of the electric field. During process crystalline cone created and the jet thrown toward the collector observed [1, 2, 5, 8, 11]. Smooth and uniform fibers with porous structure was fabricated from neat PVA. The fiber morphology did not change with addition of Ch and incorporation of silk solution in co-electrospinning process (Fig. 2b and c). The produced Ch-PVA + Silk fibrous mat was bead-free and smooth without any branching in regular shape during the process and observation showed that blended fibers get greater fiber flexibility. The fibrous mats were tightly packed and highly entangled without node formation (Fig. 2a-c). While, the mean diameter of fiber mat produced from neat PVA was 842 **±** 205 nm (Fig. 2a). The diameters of composite Ch-PVA and Ch-PVA + Silk increased at 1070 **±** 340 and 1200 **±** 321 nm respectively (Fig. 2b and c). However, statistically there was no significant difference in size distribution or average diameter of fibrous mats in different compositions (Fig. 2e, f and g). These proved that suitable concentration and ratio of polymers as well as applied voltage in agreement with previous studies [2, 5, 8, 11]. In order to achieve a good electrospun fiber scaffold, suitable concentration of polymer and solubility in solvent are vital parameters to obtain homogenous, continuous and uniform size specimen which could result lack of phase separation traces [1, 2, 5, 8, 11]. Meanwhile, the average pore sizes of PVA and Ch-PVA were 1.33 ± 0.49 and 0.9 ± 0.31 μm. The mean pore size of Ch-PVA + Silk fibrous mat was 2.2 ± 0.9 μm which indicated better pore size for permeation of oxygen and metabolites (Table 1). The Ch-PVA + Silk fiber had a porosity of 68 ± 4.4% of the total scaffold volume in comparison with 74 ± 3.9% and 52 ± 4.7% for neat PVA and hybrid Ch-PVA fibrous mats (Table 1). The high porosity of the PVA fiber is desired due to ease transfer of nutrients, oxygen and other metabolites as well as support moisture and hydrophilicity of structure. Moreover, the pore size and interconnectivity of pore network would be facilitated cellular migration and in following angiogenesis which is important in wound healing and tissue engineering. The smallest pore size and porosity of fiber mat found for Ch-PVA fiber which could be due to water solubility of polymers as well as proper selection of processing parameters [5, 8, 14, 21, 26].

**Table 1:** Characterization of produced nanofibers in different composition

Composition	Mean diameter (nm)	Pore size (μm)	Porosity (%)
**PVA**	842 ± 205	1.33 ± 0.49	74 ± 3.9
**Ch-PVA**	1070 ± 340	0.9 ± 0.31	52 ± 4.7
**Ch-PVA + Silk**	1200 ± 321	2.2 ± 0.9	68 ± 4.4

### FTIR spectroscopy

**Fig. 3:**
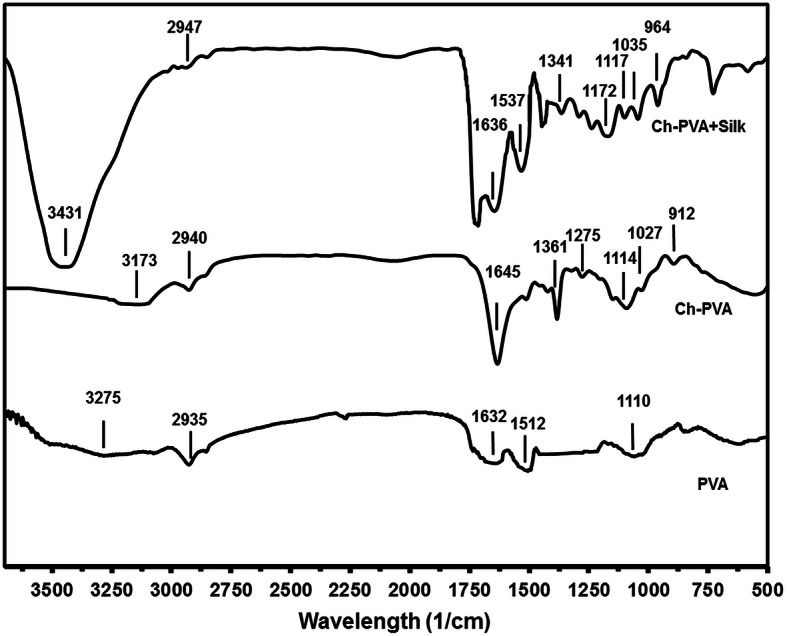
FTIR spectrum of scaffold substrates including PVA, Ch-PVA, Ch-PVA + Silk

Figure 3 showed the FTIR spectrums of the fabricated PVA fiber. The peaks of PVA were revealed by the bands 829, 1110, 1731, 2925 and 3185 cm^− 1^ for vibration stretching of C**–**C in alkyl chain back bone, C-O stretching, C**=**O stretching, C-H of alkyl stretching mode and OH vibrations respectively. The FTIR spectrum of Ch-PVA fiber mat showed a broad peak at 3338 cm^− 1^, attributed to the O-H and N-H stretching vibrations which shifted to the higher wave number with addition of Ch to PVA materials (Fig. 3). The resonance band at 1645 and 1512 cm^− 1^ related to amide I band (C=O stretching) and amide II (N-H bending and C-H stretching). Additionally, the pair peaks of 1129 and921 cm^− 1^ were arising from saccharide structure of Ch and also 1089 and 1027 cm^− 1^ were related to the skeletal vibrations of C=O stretching of the glucosamineresidues. The main characteristic absorption bands at 1636 cm^− 1^ and 1537 cm^− 1^ corresponded to amide I, C**=**O stretching and for amide II, secondary N-H bonding due to b-sheet structure in silk structure. The peaks at 1341 cm^− 1^ and 727 cm^− 1^ assigned for amide III, C-N and N-H functionalities and for amide V respectively. The created fiber structure in Ch-PVA + Silk are due to intermolecular hydrogen bonds which occur between the hydroxyl group in PVA and the -N-H/C=O bonds in grafted Ch and silk. However, noted shifts were not detected in the FTIR as there was no functional group alternation in the composite fiber. The FTIR spectroscopy could detect presence of the chemical bonds of specific polymers utilized for composite Ch-PVA + Silk fibrous mat.

### Swelling and hydrophilicity

**Fig. 4:**
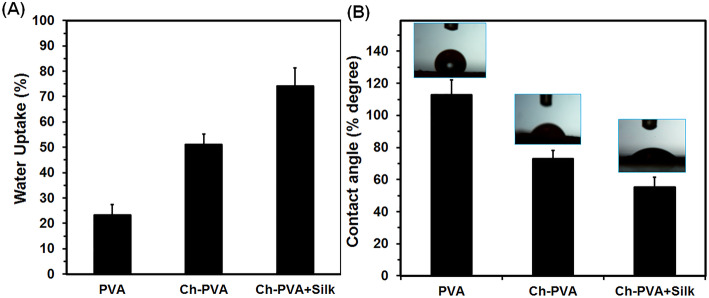
Analysis of resultant PVA, Ch-PVA, Ch-PVA + Silk fiber mats. **a** wettability, (**b**) contact angle, (Vertical bars: standard deviations; StDev, *n* = 4)

Hydrophilic/hydrophobic property of hydrogel manipulates cellular behavior including cell attachment, proliferation and migration potential through permeation of nutrient, metabolites and oxygen [5, 8, 10, 14]. Moreover, wettability of dressing area is an important element to maintain tissue moisture during wound healing process [5, 8, 10, 14]. The PVA fiber showed trivial water absorption at 21% of initial weight of fiber (Fig. 4a). The hybrid fibers of Ch-PVA and Ch-PVA + Silk absorbed water molecules up to 51 and 74% respectively which indicate addition of Ch and silk could improve swelling behavior of fibers (Fig. 4a). High water absorbency of hydrogel fibers containing Ch and silk could be due to hydrogen bonding among polymeric chains and water molecules [1, 5, 6, 10, 14, 27]. The hydrophilic functional groups in polymeric structure of Ch-PVA + Silk such as amine (NH_2_), hydroxyl [−OH], amide (−CONH-, −CONH_2_) and sulphate (−SO_3_H) would be improved swelling degree of fibers [5, 10, 14, 27]. As seen in Fig. 4b, average contact angles of the fibers decreased by hybridization pf polymers from 112° **±** 8.1° for neat PVA until 54° **±** 6.4° for hybrid Ch-PVA + Silk fibers which means higher hydrophilic characteristics of resultant fibers. The utilization of Ch and silk enhanced hydrophilic characteristic of fibrous mats owing to the presence of hydrophilic groups of Ch and silk on their structures and also Wenzel wetting model that occurs on heterogeneous surface geometries [26, 28]. The Ch and silk could circumvent poor hydrophilicity of PVA fiber and provide desirable microenvironment for cell culture, tissue engineering as well as wound healing application [5, 8, 14, 21, 27, 28].

### Mechanical property and degradability

**Fig. 5:**
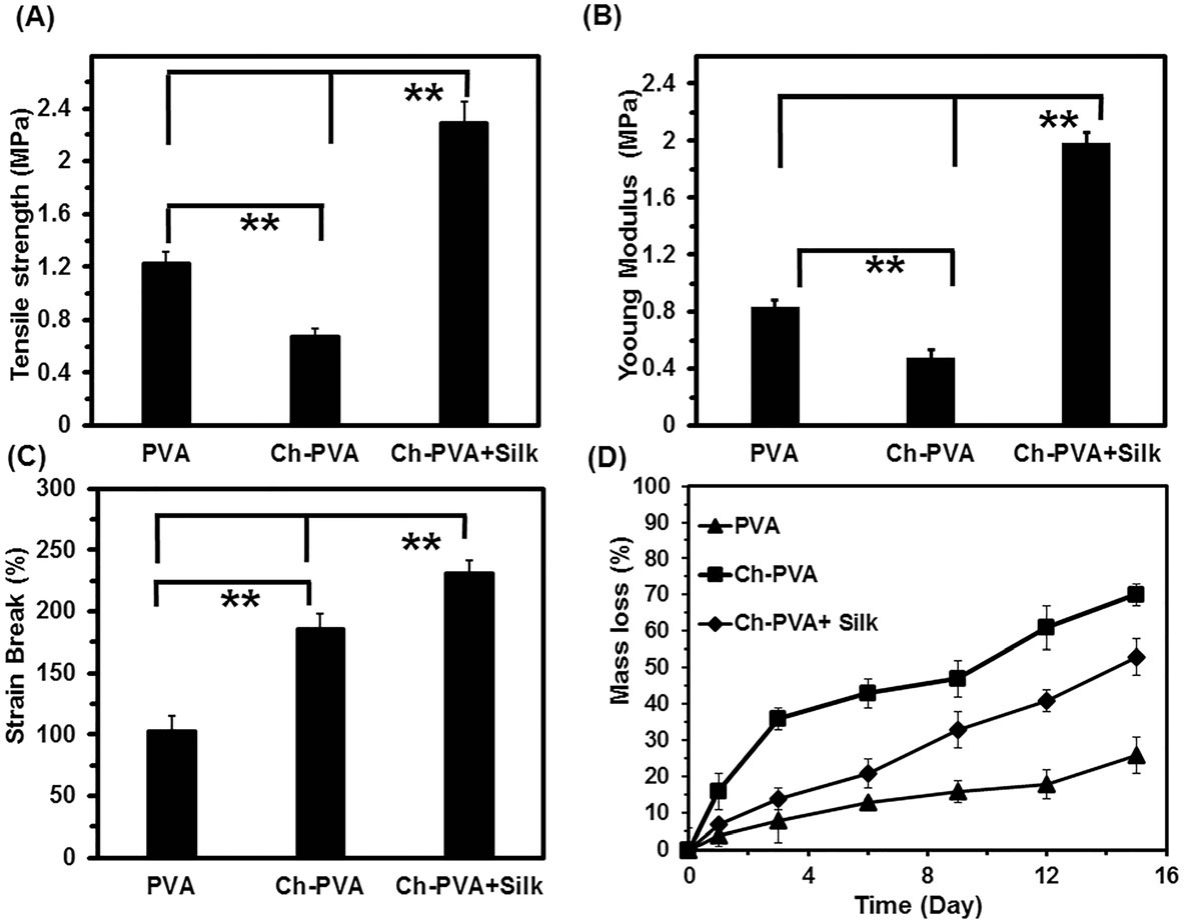
Analysis of resultant PVA, Ch-PVA and Ch-PVA + Silk fiber mats including (**a**) tensile strength, (**b**) young modulus, (**c**) strain break (**d**) in vitro degradation rate. (Vertical bars: standard deviations; SDs, *n* = 5)

Blending of PVA with Ch or incorporation of silk have an obvious impact on the mechanical properties of PVA fibrous mats (Fig. 5a-c). Mechanical strength and Young’s modulus of PVA based fibrous mat reduced by admixing Ch to the PVA solution (Fig. 5a and b). The Ch-PVA + Silk fibrous mat exhibited improvement in mechanical strength and Young’s modulus with incorporation of co-electrospinning silk and these values were more than two times of results of PVA or Ch-PVA fibrous mat (Fig. 5a and b). Although, porosity and average pore size of Ch-PVA fibrous mat was lower than PVA fiber which means tight fiber network. However, the results suggest that chemical interaction of co-electrospun silk and blended Ch with PVA could be reason of enhanced strengthening [5, 12, 20]. The PVA fiber mat was break at about 100% elongation. The breaking strain of fibers was found to increase from around 100 to 220% upon utilization of Ch and silk in composite fibrous mat (Fig. 5c). Degradation of fibrous mat is important parameter in applicability of tissue engineering scaffolds which allow cell proliferation, migration and extracellular matrix replacement during cellular growth [2, 12, 14, 19, 20, 28]. The neat PVA fiber mat showed lowest rate of weight loss compared to Ch-PVA and Ch-PVA + Silk mats (Fig. 5d). The weight loss of PVA specimen was lower than 20% until 14 days. The hybrid fibers comprising Ch-PVA and Ch-PVA + Silk mats showed an accelerated weight loss at about three and two times compared with PVA sample until 14 days (Fig. 5d). The Ch-PVA can be easily dissolved and hydrolyzed in aqueous media. Meanwhile, incorporation of silk in Ch-PVA+ Silk fiber due to highly fibroin β sheet in the silk structure resulted slower degradation compared with Ch-PVA fiber mat [10, 27]. These results would be confirmed by corresponding mechanical stiffness and hydrophilicity behavior of produced fibrous mats.

### Stem cell characterization and differentiation

**Fig. 6:**
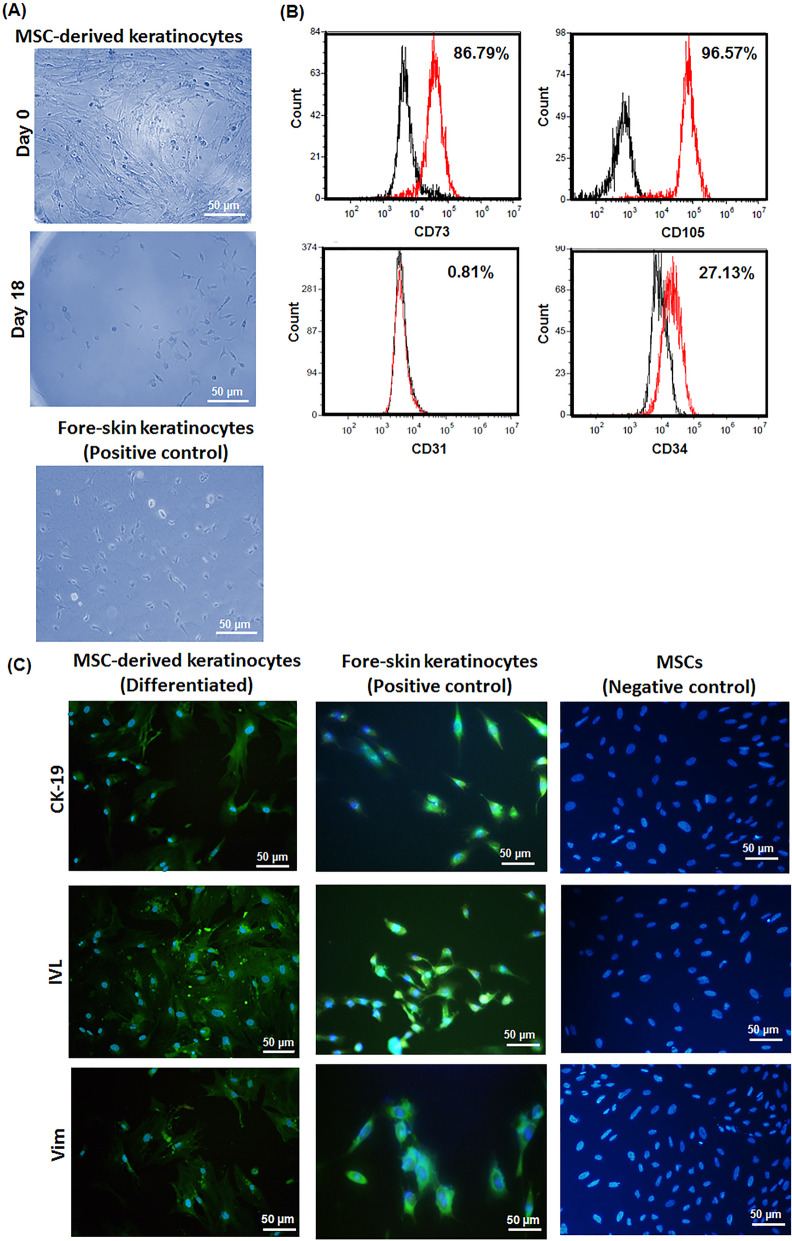
Morphology of seeded stem cells isolated from mice on day 0 and differentiated mesenchymal stem cells to keratinocyte type on day 18 in differentiating media. **b** Flow cytometry histograms of MSCs stained with fluorescein conjugated antibody. Black: without staining; red: stained cells (**c**) Expression of keratinocyte proteins including cytokeratin-19 (CK-19), involucrin (IVL) and vimentin (vim) after 18 days in differentiating media for MSC-derived keratinocytes, mice foreskin-derived keratinocytes as a positive control and MSCs in conventional culture medium. Nuclei were counterstained with DAPI

Isolated MSCs on culture dish could be adhered to dish culture and totally were spread and elongated in homogenous shape and morphology (Fig. 6a). Presence of MSC markers including CD73 and CD105 and the absence of endothelial cell marker CD31 as well as hematopoietic stem cell marker CD45 confirmed by flow cytometry analysis of the MSCs with specific antibodies Fig. 6b [3, 29–31]. Therefore, flow cytometry verified the bMSC characteristics for isolated cells. To induce keratinocyte differentiation of MSCs in long-term cultures, the isolated cells were exposed specific differentiating media [32, 33]. The MSC differentiation observed by phase contrast microscopy and immunocytochemical analysis compare with mice foreskin-derived keratinocytes as a positive control (Fig. 6a and c). The microphotograph shows evidences of polarization in the treated cells with differentiating media on day 18 and cells were in polygonal and cobblestone morphology with colonies likewise foreskin-derived keratinocytes which is specification of keratinocyte (Fig. 6a). As shown in Fig. 6c the differentiated MSCs into keratinocytes were positively expressed keratinocyte proteins including cytokeratin-19 (CK-19), involucrin (IVL) and vimentin (Vim) markers after 18 days in conditioned culture media similar results with foreskin-derived keratinocytes. It can be seen MSCs were negative to express CK19, IVL and Vim in undifferentiated media and did not expressed essential keratinocyte proteins. The utilization of calcium stimulus and EGF which bind to EGF receptor triggering signals was reliable approach for isolated MSC differentiation into keratinocytes. These data support the possibility of wider applications of MSCs for tissue engineering of skin through evidence for epidermal differentiation induction.

### Cell viability and proliferation

**Fig. 7:**
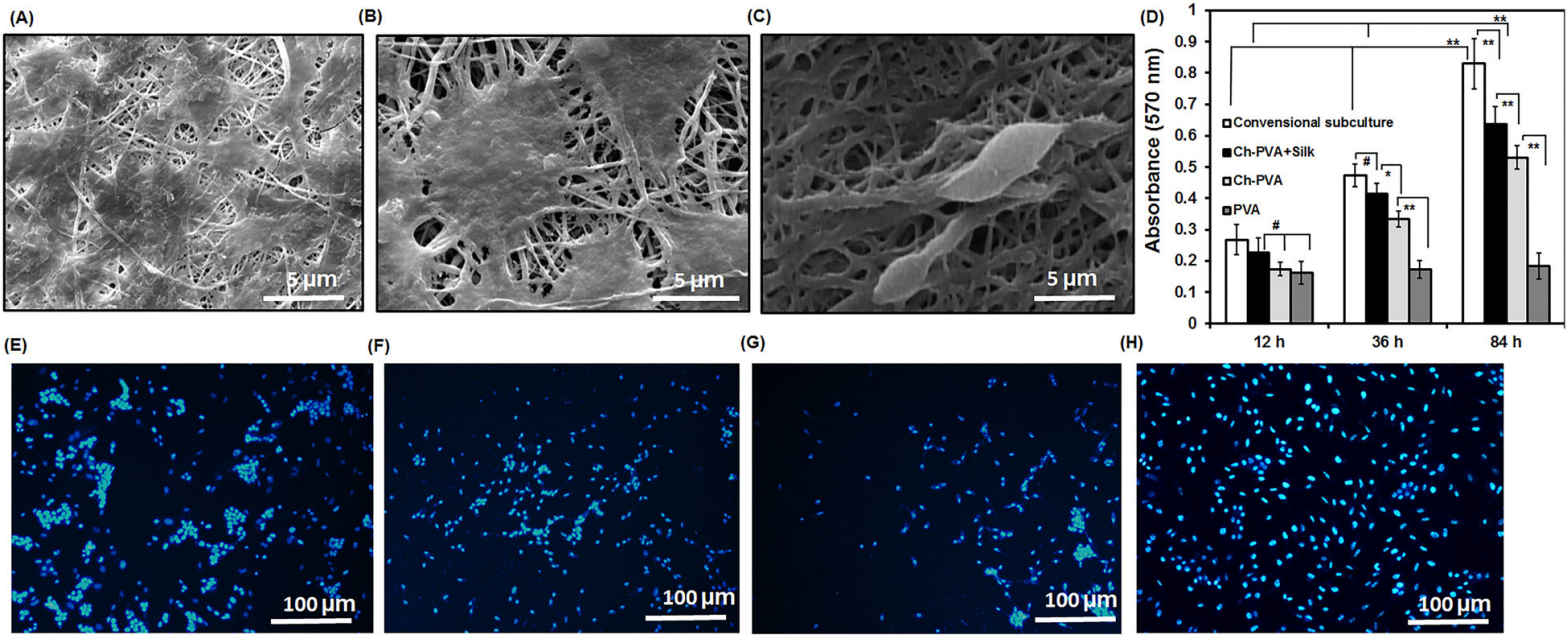
**a**, **b**, **c** SEM photograph of MSC-derived keratinocytes attachment on fiber mats after 84 h seeding. **d** Cellular growth of seeded keratinocyte in PVA, Ch-PVA, Ch-PVA + Silk fibrous mats determined by MTT. **e**, **f**, **g**, **h** Fluorescence image of the DAPI stained nuclei of differentiated MSCs to keratinocyte after 84 h seeding. **a** and **e** Ch-PVA + Silk fibrous mat, (**b** and **f**) Ch-PVA fibrous mat, (**c** and **g**) PVA fibrous mat and (**h**) conventional subculture plate (Control). (Vertical bars: StdDv, *n =* 4, *p < .05, **p < .01, and #: stand for not significant differences).

To evaluate the biocompatibility of fabricated fibrous mats, cell culture experiments were performed by seeding MSC-derived keratinocytes on samples. The cellular morphology and metabolic activity of cells detected by SEM, DAPI staining and MTT assays for seeded cells on fibrous mats as well as polystyrene tissue culture plate (PS; control). Electronic microscopy photographs showed that cells attached, grew and spread extensively with cytoskeleton extension on the Ch-PVA and Ch-PVA + Silk fibrous mats (Fig. 7a and b) which proves Ch and silk might be appropriate structure for cell culture systems [5, 34]. Whereas, the MSC-derived keratinocytes on PVA fibrous mat did not attached and were in spheroid shape (Fig. 7c). As can be seen, there were no significant differences between the cell viability of PVA and Ch-PVA fibrous mats in initial time of culture (Fig. 7d). The Ch-PVA + Silk and polystyrene tissue culture plate (PS) conditions showed higher number of attached cells in initial time of culture at 12 h. Interestingly, viability of MSC-derived keratinocytes enhanced during extend time of incubation for Ch-PVA + Silk fibrous mat and cellular growth was reached 3 times higher than neat PVA fibrous mat group (Fig. 7d). The cells could maintain their viability on PVA and Ch-PVA during extended time of incubation and lower cellular growth could be due to lack of sufficient cell adhesive ligands in these fibrous mats. However, cells seeded on PS showed higher value compared with other cell seeded fibrous mat conditions (Fig. 7d). Besides, as seen in Fig. 7e-g, composite Ch-PVA + Silk mats showed higher number of viable cells and their accumulation using dapi staining which confirmed proliferation of MSC-derived keratinocytes. Results in desired cell proliferation could be due to existence of cell recognition signals as well as hydrophilic surface tendency of Ch-PVA + Silk fibers (Fig. 4a and b) [3, 7, 16, 27, 28, 32, 35]. In summary, these data substantiated that Ch-PVA + Silk fibrous mat possessed proper cytocompatibility and incorporation of Ch as well as silk resulted increase in cell adhesion and proliferation. Hence, hybrid Ch-PVA + Silk fiber revealed high biocompatibility and potential applicability for wound healing and tissue engineering applications (Fig. 7).

### Wound closure evaluation and histological analysis

**Fig. 8:**
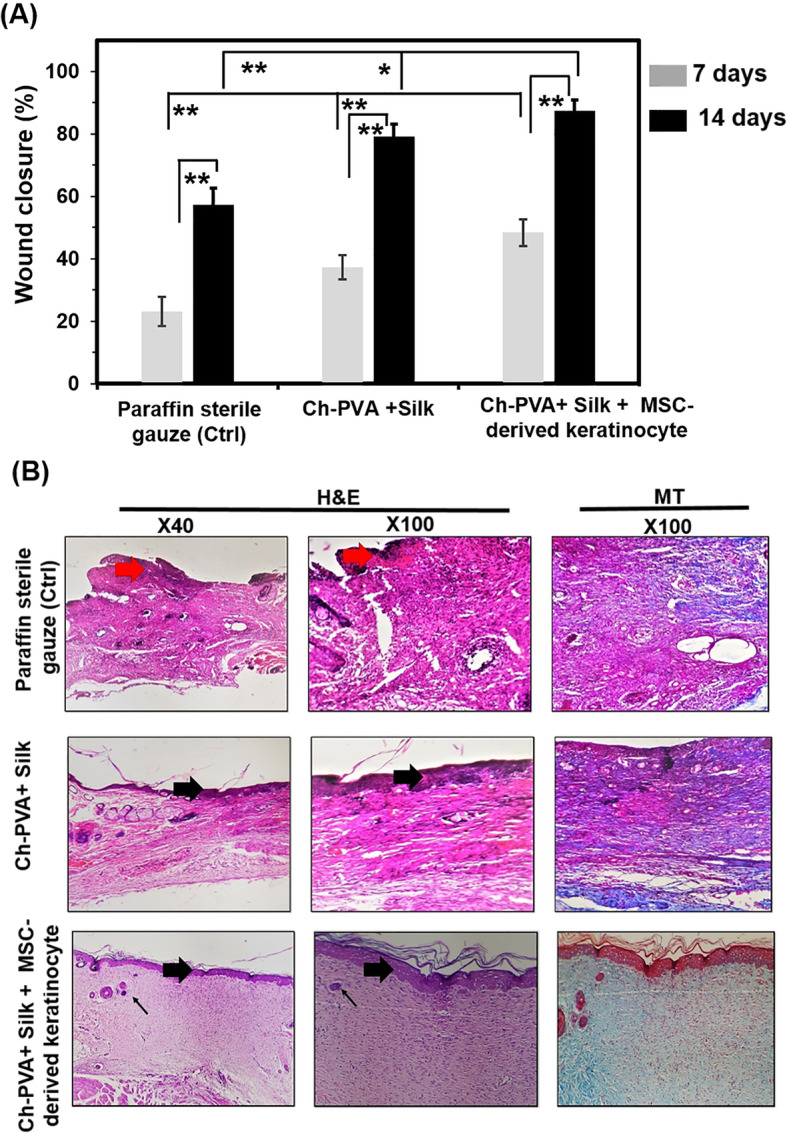
**a** Quantity of wound contraction of experimental groups at different times (**b**) H&E and MT stained microscopic sections of healed incisions at 14 days’ post-treatment, Red thick arrows: crusty scab, thin arrows: rejuvenating of hair follicles (skin appendages), Black thick arrows: re-epithelializatfion

Due to proper in vitro results of Ch-PVA+ Silk fiber mat (Fig. 7), this fiber construct was utilized for in vivo wound treating experiments. We performed in vivo wound treating experiments using paraffin sterile gauze (as a negative control; Ctrl), and Ch-PVA + Silk fiber with and without MSC-derived keratinocytes for 14 days. The wound sizes show treatment-dependent and time-dependent differences in rate of wound contractions as indicated in Fig. 8a. The wound closure measurements indicated significant wound healing after 7 days treating by Ch-PVA + Silk fibrous mat with and without MSC-derived keratinocytes compared to paraffin sterile gauze condition (Fig. 8a). The decrease in size of the wound area in comparison to its original size was 87.4 ***±*** 3.4% for Ch-PVA + Silk fibrous mat with MSC-derived keratinocytes, 79.1 ***±*** 4.1% for Ch-PVA + Silk fibrous mat while it was only 57.2 ***±*** 5.4% for the paraffin sterile gauze condition at the 14th day. High degrees of wound healing suggest that the Ch-PVA + Silk fibrous mat due to worthy moisture condition and distinctive hemostatic properties could actively promote wound healing process with higher degrees of re-epithelialization [16, 19, 20, 27, 28, 32].

Histological analysis of the wounds in the paraffin sterile gauze group displayed evident leukocyte infiltration, granulation tissue formation, and a crusty scab, however, epidermal layer has not been formed (Fig. 8b). Histopathological evaluation of Ch-PVA + Silk group showed moderate infiltration of inflammatory cells into the wound area (Fig. 8b). Although, the epithelialization process was initiated, this process was incomplete in all samples in this group and the epithelial layer was thinner than normal skin. The inflammatory cells were significantly reduced at Ch-PVA + Silk group in comparison to paraffin sterile gauze group. Besides, we did not observed wound infection for treated conditions. It is expected that the antimicrobial effect of Ch due to destabilization of the outer membrane of gram-negative bacteria and permeabilization of the microbial plasma membrane be the reson for obtained results [3, 29, 36–39]. However the antibacterial effect and possible mechanism of the combination therapy is required to study through agar plate method and bacteriostatic ring test [38, 40].

Histopathological evaluation of the wounds treated by Ch-PVA + Silk fiber with MSC-derived keratinocyte group showed a considerable reduction of inflammatory cells in comparison to the other groups (Fig. 8b). A complete epithelial layer with the presence of rete ridges was formed in Ch-PVA + Silk fibrous mat containing cells. This group showed more resemblance to normal skin, with a thin epidermis (normal thickness of skin layers), presence of normal rete ridges, rejuvenation of the hair follicles (skin appendages). It seems that this treatment showed the best results while compared to other experimental groups. It is reported that keratinocytes play a vital role in epidermal restoration during wound healing through proliferation, migration and re-epithelialization. The differentiated MSC to keratinocyte would implement its impact through provision of structural support, protection of epithelial cells from mechanical and non-mechanical stress and the regulation of apoptosis and protein synthesis [23, 40]. The wound healing process is heavily dependent on collagen synthesis. Therefore, to further investigate the effect of different treatments on wound healing, sections of animal skin tissues were stained with MT staining. This staining was used to recognize the progress of collagen synthesis during granulation tissue (GT) formation and matrix remodeling. The collagen fibers were stained blue-green in MT staining method which the intensity of this color corresponds to the relative amount of deposited total collagen and reflects the advancement of collagen synthesis and remodeling [13, 14, 16, 33, 36]. The results indicated that among the experimental groups, the Ch-PVA + Silk with MSC-derived keratinocyte group had the greatest collagen synthesis. On the other hand, the rate of collagen fiber synthesis and deposition in wound were the lowest in paraffin sterile gauze group.

### Histomorphometric analysis

The histomorphometric analysis was done and the results have been presented in Tables 2 and 3. Amongst all groups, re-epithelialization in control groups was minimum and it was mostly filled with immature GT formation. The best re-epithelialization was seen in the Ch-PVA + Silk + MSC-derived keratinocyte treatment group. Moreover, the total number of inflammatory cells in Ch-PVA + Silk + MSC-derived keratinocyte treatment group was significantly reduced in comparison with others *(P-value* ***<*** *0.01)*. Overall, the healing results of Ch-PVA + Silk + MSC-derived keratinocyte treatment group was more similar to that of the normal skin with normal thickness of epidermal layer and rejuvenation of the hair follicles and other skin appendages.

**Table 2:** Histomorphometric analysis of different experimental groups

Groups	Epitheliogenesis Score
Ctrl	0
Ch-PVA + Silk	1
Ch-PVA + Silk + MSC-derived keratinocytes	4

**Table 3:** Histomorphometric analysis of wounds

Groups	Inflammatory cells	Blood vessels	Collagen content
Ctrl	84.6 ± 9.0	26.2 ± 4.1	23 ± 5.1
Ch-PVA + Silk	49 ± 5.5**	19.7 ± 2.5	31.2 ± 2.8*
Ch-PVA + Silk + MSC-derived keratinocytes	13 ± 2***	6.2 ± 1.7***	79.7 ± 2.9***

*, **, ***: values indicate treatment group versus un-treatment group (empty control); ** *P-value* < 0.01, ***P-value < 0.001

## Conclusion

This study was designed to develop hybrid fiber mat compose of Ch, PVA and silk through co-electrospining process to evaluate their synergic effect on wound healing process. The physical and biochemical specification of fabricated hybrid fibrous mat were investigated. The effect of composition on structure, morphology, hydrophilicity and mechanical properties of the fibers were also studied. The Ch-PVA + Silk hybrid fiber prominently showed superior mechanical properties and desire swelling as well as hydrophilic microenvironment to those of pure PVA and Ch-PVA fibers. Incorporation of blended Ch and co-electrospun silk in PVA based fibrous mat presented excellent cell adhesion and proliferation in comparison to the neat PVA and Ch-PVA fibers. The in vivo study showed that composite Ch-PVA + Silk fibrous mat in presence of MSC-derived keratinocytes could stimulate wound healing and skin tissue regeneration. These components are structurally and morphologically suitable for stem cell culture and application in tissue engineering because of their biophysical properties and cytocompatibility.

## Data Availability

The datasets used and/or analyzed during the current study are available from the corresponding author on reasonable request.

## Abbreviations

PVA: Polyvinyl alcohol
Ch: Chitosan
ECM: Extracellular matrix
CMC: Carboxymethyl cellulose
HA: Hyaluronic acid
FDA: Food and drug administration
Ala: Alanine
Gly: Glycine
Ser: Serine
Asp: Aspartic acid
GAG: Glycosaminoglycan
DAPI: 4′,6-diamidino-2-phenylindole
MSCs: Mesenchymal stem cells
CK-19: Cytokeratin-19
IVL: Involucrin
Vim: Vimentin
MTT: 3-[4,5-dimethyl-2-thia-zolyl]-2, 5-diphenyl-2H-tetrazolium bromide
GT: Granulation tissue
MT: Masson’s trichrome
H&E: Hematoxylin and eosin
EGF: Epidermal growth factor
KGF: Keratinocyte growth factor
